# Between Care and Coercion: Asylum Seekers’ Experiences With COVID-19 Containment and Mitigation Measures in German Reception Centres

**DOI:** 10.3389/ijph.2023.1605230

**Published:** 2023-03-13

**Authors:** Eilin Rast, Clara Perplies, Louise Biddle, Kayvan Bozorgmehr

**Affiliations:** ^1^ Section Health Equity Studies & Migration, Department of General Practice and Health Services Research, Heidelberg University Hospital, Heidelberg, Germany; ^2^ Department of Population Medicine and Health Services Research, School of Public Health, Bielefeld University, Bielefeld, Germany

**Keywords:** mental health, qualitative research, COVID-19, quarantine, refugees

## Abstract

**Objectives:** COVID-19 containment and mitigation measures have been criticised for amplifying pre-existing individual and structural vulnerabilities among asylum seekers. We qualitatively explored their experiences with and attitudes towards pandemic measures to inform people-centred responses in future health emergencies.

**Methods:** We interviewed eleven asylum seekers in a German reception centre (July-December 2020). The semi-structured interviews were recorded, transcribed, and analysed thematically with an inductive-deductive approach.

**Results:** Quarantine was experienced as burdensome by participants. Shortcomings in social support, everyday necessities, information, hygiene, and daily activities exacerbated the strains of quarantine. Interviewees held different opinions about the usefulness and appropriateness of the various containment and mitigation measures. These opinions differed by individual risk perception and the measures’ comprehensibility and compatibility with personal needs. Power asymmetries related to the asylum system furthermore impacted on preventive behaviour.

**Conclusion:** Quarantine can amplify mental health burdens and power asymmetries and can therefore constitute a considerable stressor for asylum seekers. Provision of diversity-sensitive information, daily necessities, and accessible psychosocial support is required to counteract adverse psychosocial impacts of pandemic measures and safeguard wellbeing in this population.

## Introduction

Crowded living conditions in shared accommodations, like reception centres (RCs) for asylum seekers, have increased the risk of infection with SARS-CoV-2 (1–5). Outbreaks in RCs across Europe have been reported from early on in the pandemic (2, 3, 5, 6). To curb the spread of the virus, different preventive measures were implemented in collective accommodations for asylum seekers throughout Europe. Mitigation measures included physical distancing, hygiene measures, health information, and the re-organisation of services, while containment measures included, for example, screening asymptomatic individuals for infection, and quarantine for infected individuals and contact persons (7–9). In addition to quarantine for detected cases, quarantine for individuals entering national asylum systems was common practice across European countries (7). Germany has held the largest share of asylum applications in Europe in the past years (121,955 in 2020) (10). Universal screening for SARS-CoV-2 (*via* PCR-testing) and a fourteen-day quarantine for all new arrivals (independent of screening results) were widely implemented throughout the country (11). Furthermore, mass quarantine—defined as indiscriminate restrictions of within and in-and-out movement for all inhabitants of collective accommodations—was recurrently implemented in Germany and other countries (1, 5).

However, the psychosocial consequences of pandemic measures, which primarily aim to safeguard physical health, need to be considered as well. It is known that quarantine and isolation can negatively impact mental health in general (12–15). For asylum seekers in particular, the COVID-19 pandemic has been regarded as an amplifier for psychological burdens that already affect this group disproportionately (5, 16–19). Empirical studies pointed to challenges and unintended consequences of implementing pandemic measures in RCs: A study among German authorities underlined the challenges of physical distancing in shared accommodations for asylum seekers and the importance of multilingual, personal communication to counter misinformation and anxiety among residents (11). Qualitative insights from Italian RCs indicated a strong focus on confinement and lockdown measures, leaving psychosocial implications largely unaddressed, although the precarious living and working conditions also impeded inhabitants from following regulations (6).

The impacts of the pandemic have been studied from the viewpoint of professionals or authorities (6, 11), but direct engagement with the perspectives of asylum seekers living in RCs remains scarce in COVID-related research. With this qualitative study, we aim to contribute to the development of people-centred pandemic responses that account for different lived realities. We sought to explore (i) how asylum seekers in RCs experience COVID-19-related measures, and (ii) what shapes their attitudes towards pandemic measures and their individual preventive behaviour.

## Methods

We conducted ten semi-structured in-depth interviews with eleven asylum seekers living in a RC in Southern Germany. By engaging with their perspectives, we aimed to generate insights into both the implementation of preventive measures and the ways in which individuals assign meaning to their experiences. In addition to formal interviews, our observations and interactions with inhabitants and staff were documented in field notes. Data collection and analysis were carried out by ER and CP.

The terms asylum seeker and refugee are often used interchangeably. Legally speaking, an asylum seeker is an individual whose asylum claim has yet to be processed and who thus has not yet been granted international protection as a refugee (20). We use asylum seeker throughout this paper due to our focus on RCs (which mostly accommodate asylum seekers), but note that these measures affected individuals at various stages of the asylum process.

### Setting and Sampling

To capture the experiences and attitudes towards both mitigation and containment measures, we aimed to include asylum seekers with quarantine experience in the study. We therefore selected a large RC (approximately 650-800 inhabitants at time study) as a field site, where, apart from the routine quarantine upon arrival (i.e., mandatory for all asylum seekers, regardless of PCR-test results), a mass quarantine had been implemented some months prior to our study. Mitigation measures applied by the authorities included health information, hygiene measures, and physical distancing. Group activities usually offered at the RC, e.g., language classes or play groups for children, had been suspended and the canteen closed. Instead, meals were distributed by car for consumption in the living areas. Primary medical care continued to be available at the on-site outpatient clinic.

Participants were selected purposively: Apart from quarantine experience, we aimed for diversity in the sample regarding sex, nationality, and family situation. Recruitment was limited to adults speaking English, French, or German to enable direct communication with the field team.

We interviewed nine individuals and a married couple. Ten interviewees had entered the asylum system only recently and had undergone mandatory COVID-19 screening (none of the participants had been tested positive) followed by a fourteen-day quarantine (independent of test results). Some interviewees had undergone these measures in several RCs because of dispersal procedures in the German asylum system. The findings therefore reflect experiences from six different accommodations, which can be considered typical German reception facilities. At the RC observed, quarantine took place in guarded and fenced apartment blocks, where asylum seekers were either housed alone, with other individuals, or their families (some including children). One participant had—after the detection of confirmed SARS-CoV-2 cases in the accommodation—undergone a more than two-week mass quarantine, during which the inhabitants were allowed to move freely within the RC’s premises wearing face masks, but not to leave it.

### Data Collection

Data collection took place during two epidemiologically different periods in the early phase of the COVID-19 pandemic: Six interviews were conducted in July and August 2020, when the average weekly incidence rate in Germany per 100,000 was 7.1 and cases were often travel-associated (21). In this phase, asylum seekers undergoing the fourteen-day quarantine for new arrivals were invited to participate in the study and interviews were conducted face-to-face and jointly by ER and CP in the participants’ living areas during quarantine. In response to the changing pandemic situation, the fluctuating numbers of individuals in quarantine, and temporary restrictions of field access, the recruitment strategy for the remaining interviews was adjusted in November and December 2020 during the second COVID-19 wave in Germany (21). During this time, potential participants were approached in the waiting area of the on-site outpatient clinic or through pre-existing contacts of the research team. These interviews were conducted by one researcher ER in the clinic and in one case by telephone to account for tightened contact restrictions.

The interview guide (see Supplementary File S1) was drafted by ER and CP and then revised together with the other co-authors to ensure that interviewees have enough room to share their perspectives while including all relevant aspects of disease prevention and control. The final interview guide covered processes and wellbeing during quarantine, COVID screening, information on SARS-CoV-2, and experiences with and the rating of mitigation measures. The conversations were audio-recorded and lasted 50 min on average. One of the ten interviews was conducted with a couple. In three others, flatmates or spouses were present by request of the participants without actively participating. Informed consent was obtained prior to all interviews. Guidelines on the prevention of COVID transmission were respected throughout data collection.

### Analysis

All interviews were transcribed verbatim including paralinguistic elements. Transcripts and field notes were analysed thematically in their original language (English, French, or German) to avoid loss of meaning through translation. After familiarising with the data, a preliminary coding scheme was developed, containing deductive codes related to the research questions and inductive codes derived from the data. Following a first round of coding with MaxQDA version 20, codes and sub-codes were further refined in an iterative process through recurring discussions (see Supplementary File S2 for coding scheme). Finally, central themes were generated and relations between themes and subthemes were explored.

### Reflexivity

We understand our research process as contextual, situated, and contingent on our own subjectivity as researchers (22, 23), which demands positioning. All authors are familiar with the study field through previous research and extensive exposure to asylum contexts. We are conscious of our socially privileged situation and the empirical and normative limits of “representing” the voices of our research subjects (24). Research diaries and team discussions fostered reflections on power dynamics and ethical aspects of the study and allowed to review interpretations and to include multidisciplinary perspectives.

## Results

Based on the interviews and field notes, the following main themes were identified in the analysis: mental health and daily needs during quarantine, preventive behaviour, and the role of power asymmetries in the experience of the pandemic measures. Individual attitudes towards the measures surface throughout these sections. The sociodemographic characteristics of the 11 participants are displayed in Table 1.

**TABLE 1:** Sociodemographic characteristics of participants (N = 11). Germany, 2020.

Legal status	Asylum seekers: 10
	Asylum application rejected/expulsion temporarily suspended: 1^a^
Age in years	18–19: 2
	20–29: 3
	30–39: 4
	40–50: 2
Gender	male: 8
	female: 3
Nationality	Syria: 3
	Yemen: 1
	Algeria: 3
	Gambia: 1
	Cameroon: 1
	Tanzania: 1
	Bangladesh: 1
Educational level	No formal education: 1
	School education: 5
	University degree: 3
	Unclear: 2
Stay in German reception centres	≤14 days: 6
	2–4 weeks: 4
	≥1 year: 1
Marital status^b^	Single: 6
	Married: 5

^a^ At the time of the interview, this participant’s legal status had changed to suspension of deportation, but for easier readability, we will nevertheless refer to all interviewees as asylum seekers.

^b^ All single persons travelled alone, the married ones together with their spouses. Three couples were accompanied by their children.

### Mental Health in Quarantine

Most interviewees perceived the quarantine as lengthy and difficult. Commonly associated feelings were boredom, loneliness, sadness, and low mood. Participants described dealing with memories of war, persecution, and flight as well as worries about relatives and their own future as burdensome. In some cases, obsessive thinking and sleeping problems were reported:

*“When I stay alone in my room, I didn't sleep […] I thinking in bad think you know? That why I'm here, why my country like that? Whywhywhywhywhy. Maybe it's fucking my psychology”* (Int02).

The constrained freedom of movement within the fenced and guarded housing blocks made participants feel *“caged”* (Int10), evoking comparisons with imprisonment in most interviews. Two participants (Int03, Int06) described this as particularly stressful for individuals who had been in prison in the past.

The varying types and extent of information participants had received about the quarantine’s rationale and procedure impacted their experience of the measure. Several participants, while pointing out the strains of quarantine, recognised its aim of health protection:

*“It is hard but in another side you think about Corona virus and, to save the anybody. And then you tell yourself ‘It´s okay, I can do it.’”* (Int04).

In a few cases, quarantine combined with testing contributed to feeling “[…] comfortable and safe because when I get out [of quarantine], yes I know all people in this camp they making a test for Corona virus, they was in quarantine” (Int01). However, if the reasons of quarantine upon arrival were not clear, it appeared as an unnecessary burden:

*“I am negative. And why he put me more in quarantine. I just want know this. Why?“* (Int02).

One participant was puzzled when he learned that his quarantine would continue for another 12 days after being tested negative:

*“They should prepare you mentally […] you don’t expect that”* (Int06).

To cope with mental distress during quarantine, interviewees resorted to various strategies, including phone conversations, interactions with others in quarantine, physical activity, and focusing on the quarantine’s foreseeable end. One participant with high mental distress recounted having left quarantine temporarily for distraction and regaining a sense of freedom. In quarantine, possibilities for activities and distraction were strongly limited and contingent on whether people were accommodated alone or with others and the availability of a mobile phone and internet connection:

*“With the things you have gone through for arriving here […] psychologically it’s not easy, staying alone – like me, I don’t even have a phone”* (Int06).

This participant also mentioned the need for more proactive support during quarantine:

*“Support measures are really needed, maybe social workers that can sometimes come to check on you. There are individuals who are super stressed”* (Int06).

### Daily Needs During Quarantine

Digital communication with friends and relatives proved not only important for emotional support, but also for meeting other needs, e.g., the recharge of phone credit or supply with hygiene products, cigarettes, or special foods. The possibility to obtain practical support and daily necessities from staff was experienced heterogeneously by interviewees, some of whom also pointed out differences between accommodations. In some RCs, personnel regularly collected requests for individual needs. Elsewhere, however, participants were unaware of services available, experienced language barriers when seeking support, stopped asking for favours if staff was irresponsive, or instead sought the support of neighbours or security guards.

*“We cannot go to shop, we said to people who drive the car lunch and they said: ‘Oh, we are busy today, tomorrow’ and like this and they didn`t shopping”* (Int01).

Some participants were content with the simple fact of being provided with food, others uttered dissatisfaction about the lack of variation and low quality, because “what they give we have to eat. We […] cannot go outside” (Int07). Furthermore, inadequate hygiene conditions led to a feeling of abandonment and fear of infections for some:

*“One toilet, no hygiene – I can`t even give you the picture, you would vomit, and then we ask ourselves: You`re running away from Corona, and you come and get infectious diseases in here”* (Int10).

Apart from improvements in food and hygiene, participants suggested further ways to enhance wellbeing in quarantine: Stable Wi-Fi would not only offer multilingual entertainment, access to information, and language learning, but also enable contact with friends and family, and thereby a connection to the outside world. Parents wished for toys for their children. Also, a higher sensitivity for individual needs by staff and earlier notice about medical or administrative appointments was requested:

*“They come only for their business. They never ask us ‘[…] Do you face any problem? How can I help you?’ but how the process will go, what is your next steps, not before that day”* (Int07).

### Preventive Behaviour

While all participants stated that protection from SARS-CoV-2 was important, opinions about the general mitigation measures and individual preventive behaviour varied.

Overall, participants were familiar with the hygiene and distancing measures. The information provided by RCs was described as ranging from merely brief oral instructions, over written information, to repeated individual advice. But none of the participants declared a personal need for further information on mitigation measures, since “it is a looong time already spent in the Corona situation” (Int07).

Individual risk perception differed. For some participants, the virus was a “very dangerous disease” (Int07) imperilling their own and others’ health. Great fear was expressed by a pregnant woman whose son suffered from asthma:

*“If he gets COVID it would be a disaster. And me, I am pregnant”* (Int09).

Some others, in contrast, considered only specific groups, e.g., elderly people, as vulnerable. They were confident that their own immune system can handle the virus “like normal flu” (Int10).

In a few cases, preventive behaviour went beyond official guidelines (e.g., emphasising bodily or room hygiene). Some conscious violations of regulations were also reported, e.g., contacting people from outside of quarantine for obtaining daily necessities, or if the risk of infection among individuals in quarantine was considered low:

*“I know they […] making test COVID-19 and I […] was negative so I didn`t have any problem if I contact with them without a mask”* (Int01).

Overall, participants who disagreed with certain measures nevertheless stated that they adhered to them due to perceived social expectations, e.g., by keeping distance or wearing masks:

*“I must put a mask, I know, maybe I have different like things [opinion] about COVID-19, but this doesn`t mean I didn`t like care about other people”* (Int01).

### Power Asymmetries

Apart from risk perception and compatibility with needs, the adherence to the different measures was also influenced by power relations within the asylum system. Quarantine was regarded as simply unavoidable, “for you get in this country asylum, you have to stand it” (Int02). Some participants had the impression that, as asylum seekers, they were not allowed to question the implementation of quarantine out of fear of negative consequences:

*“I’m only an asylum seeker, […] that’s why I told you that I hope that what I am going to talk about [in this interview] won’t cause any trouble for my procedure and all that, because you are in the registration process, they might say ‘that guy talks a bit too much’”* (Int06).

One participant strongly criticised the fencing of quarantine and harsh rebukes from security personnel on preventive measures and pointed out that most asylum seekers were “humble” (Int10), not knowing their rights:

*“Mostly every asylum seeker is afraid because, if he does something wrong, ‘I might not get my papers, I might be thrown out’, so that`s the mentality of an asylum seeker who doesn`t know his rules and regulations and everything!”* (Int10).

## Discussion

This qualitative study explored asylum seekers’ perspectives of COVID-19 measures in German RCs and thereby gives a rich and intimate account on the lived experiences with public health interventions in this setting. Quarantine overall constituted a considerable stressor in this sample, while the results indicate that the measure can also contribute to a feeling of safety for individuals living in RCs provided its rationale and aim are clearly communicated. Lacking psychosocial support, material supplies, information, and activities exacerbated the strains of quarantine. The participants of this study perceived the various containment and mitigation measures on a continuum between care and coercion which depended on four main aspects: the information interviewees obtained, their individual risk perception, the compatibility of measures with personal needs, and the extent to which power asymmetries of the asylum context were amplified.

The quarantine-induced mental distress (12, 13) interacted with pre-existing psychological burdens related to participants’ flight experiences and their uncertain living situation, supporting other studies with asylum seekers that report the reactivation of traumatic experiences during quarantine (6, 16). Evidence-informed recommendations suggest that having a daily routine, digital social contact, and other meaningful activities are important to prevent negative psychological effects of quarantine and confinement (14, 25). In our study context, such coping strategies were strongly limited, especially if interviewees were housed alone, lacked digital means for social contact, or confronted language barriers. Positive psychological effects of quarantine, such as increased freedom and privacy, are rarely reported in other studies (12) and hardly applicable to RCs (26).

Participants reported that SARS-CoV-2-related information varied in quality and quantity, indicating that not all inhabitants are reached adequately by health communication activities of facilities (11). Although no interviewee declared a personal need for further information on the virus and mitigation measures, this cannot be generalised to all asylum seekers and refugees, as language barriers and internationally differing and dynamic policies can make grasping local regulations overwhelming. Furthermore, the need to counter anxiety, misinformation, and trivialisation through personal information has been emphasised by authorities responsible for asylum seeker and refugee accommodations (11). In our sample, a clear informational gap regarding the quarantine for new arrivals and resulting distress became evident. Studies from other contexts confirm the stress and confusion caused by inadequate information on quarantine and underline the need for clear communication (14).

However, comprehensible communication does not guarantee coherency in measures: participants of this study remarked discrepancies between hygiene recommendations and housing conditions. This may not only result in discomfort and perceptions of neglect, but also in mistrust of communication campaigns—an effect reported for the contradictions between physical distancing recommendations and crowded living conditions in RCs (6). A review on disease containment measures in shared accommodations for asylum seekers showed that following hygiene standards and physical distancing are common challenges in these settings (8).

This study furthermore revealed the synergies of COVID-19 and immigration policies which emphasise the power asymmetries in the asylum context. Forced migration in general can be an experience of disempowerment and status loss (27, 28). Asylum seekers in RCs are subjected to regulations affecting central areas of life (e.g., housing, work, medical care) (29, 30), that create an ambivalent simultaneity of care and control (31). With fencing and security guards enforcing quarantine and mitigation measures, control mechanisms are further increased during the pandemic, which evoked metaphors of imprisonment in the interviews. Such an enforcement of quarantine has also been reported by German authorities (11). In the present study, asylum seekers had to rely substantially on external support during quarantine. While in some RCs, effective supply systems have been implemented, others were lacking low-threshold support structures. Due to their legally vulnerable situation, demanding help or uttering critique might be avoided by those individuals fearing negative implications for their asylum procedure. Thus, the power asymmetries in the asylum system can have a silencing effect, especially on asylum seekers unsure about their rights or those experiencing language barriers. Instances in which participants’ requests were not responded to by staff illustrate that this dependency is highly problematic and can further deteriorate wellbeing, as also reported for other population groups (14). In this respect, pandemic measures operate at a thin line between protecting individuals and being an expression of care, or contributing to further “othering” (32), understood as a social categorisation practice that alienates, disempowers and stigmatises collectives vis-à-vis the more powerful, designating actors (27). We argue that the question whether power asymmetries are amplified, or adequately considered and eventually even reduced during an emergency response, is key to evaluation of pandemic responses and their relation to othering and overall wellbeing and health. Where such pre-existing asymmetries are exacerbated, public health measures (even if well-intended) may easily tip over to coercion and to unintended negative consequences for health and social wellbeing.

Our findings underline the significance of the official guidelines from the European Centre for Disease Prevention and Control (ECDC) (5) and the Robert Koch-Institute (RKI, Germany’s public health agency) (17), which were issued specifically for RCs prior to our data collection. They contain evidence-informed recommendations on testing, hygiene, distancing, and quarantine measures. They also advise the adequate provision of material supplies during quarantine, health information that accounts for different language and literacy competencies, usage of interpreters to enable bidirectional communication, and frequent cleaning of facilities. Moreover, engagement of inhabitants should be preferred over top-down enforcement of measures. The guidelines further emphasise that mental health needs must be addressed and stigmatisation and discrimination of asylum seekers avoided. Our results show that these guidelines were not universally implemented until December 2020, i.e., up-to 6 months after their release. Deviations from the ECDC and RKI recommendations, which contributed to the deterioration of the inhabitants’ wellbeing, were particularly visible with respect to the following principles: adequate provision of daily necessities and social support, avoidance of fencing and securitisation of quarantine, as well as communication strategies which involve interpreters to overcome language barriers and to ensure that health information is understood and needs can be adequately articulated.

In view of the discrepancies between official guidelines and locally implemented measures, the question arises, which factors affect decision making processes under the uncertainties of a public health crisis such as the pandemic. Further research is needed to understand how public health interventions during the pandemic are influenced by othering, e.g., in the reception system. Processes of othering have been reported with regard to public health interventions targeting asylum seekers, who frequently have been portrayed as “carriers of disease” (27) in receiving countries. The current pandemic yields further examples of contested measures: Mass quarantine and curfews exceeding those implemented in the host population have been reported for asylum seekers in Europe (2, 6, 33). In German RCs, quarantine for new arrivals was maintained between the first two COVID-19 waves, at a time when quarantine upon entering Germany was replaced by testing (21). Since such discriminatory measures potentially deteriorate mental health and overall wellbeing, restrict individual freedom, and expose individuals to a higher risk of infection (34), further research is needed to uncover the underlying rationales of public health interventions in the asylum context, and their potentially re-enforcing effects on discriminatory perceptions and behaviour towards asylum seekers.

### Limitations

The study’s limitations pertain to the relatively small sample, which mostly consists of newly arrived asylum seekers, does not include single women, and was constrained by three languages. Even though we aimed to capture experiences with a variety of pandemic measures, a focus on the quarantine for new arrivals emerged due to its topicality for the participants at the time of the interviews. Perspectives on measures are likely to change with the course of the pandemic and altering living conditions. Our findings can therefore not be generalised to asylum seekers and refugees in other life situations. Furthermore, socially desirable statements and conduct by the participants might have been evoked by our own adherence to preventive measures during the interviews. Also, it is possible that during recruitment, individuals hesitant to share their views (e.g., because of their structural vulnerabilities) did not take part, which means that crucial perspectives might have been excluded from the study. Due to the focus on the perspectives of newly arriving asylum seekers in RCs, the experiences of asylum seekers and refugees in other contexts remain unaddressed by our study. As pandemic measures impact the access to employment, education, and social services, the effects on asylum procedures and societal integration need to be assessed.

### Conclusion

In this study, quarantine—in combination with the contextual factors of RCs—overall amplified the strains associated with flight and asylum seeking, such as psychological burdens and dependency relations. Nevertheless, its rationale of health protection was recognised by most participants. The varying individual attitudes towards containment and mitigation measures were contingent on their comprehensibility, coherency, and compatibility with personal needs. Adverse psychosocial side-effects of pandemic measures must be minimised in line with existing national and international guidelines by ensuring adequate living conditions, pro-active support structures, as well as diversity-sensitive information.
