# Supplementary material for: Between Care and Coercion: Asylum Seekers’ Experiences With COVID-19 Containment and Mitigation Measures in German Reception Centres
Source: Int J Public Health. 2023 Mar 13;68:1605230. doi: 10.3389/ijph.2023.1605230 (PMC10041458; doi:10.3389/ijph.2023.1605230)
Supplement: Supplementary file 1 [file DataSheet2.docx]

# Supplementary file 2 for the original article:

**“Between Care and Coercion: Asylum Seekers’ Experiences With COVID-19 Containment and Mitigation Measures in German Reception Centres”**

Published in the special issue “Migration health around the globe – a construction site with many challenges” of the International Journal of Public Health

Coding Scheme. Germany, 2020

| **List of Codes** |
| --- |
| **Context/Setting** |
| Asylum procedure |
| Living/housing conditions |
| Personal living situation before arrival at the reception centre |
| Other |
| **Health care** |
| Regular medical care |
| Health screening for COVID-19 symptoms |
| Medical check-up, vaccination as part of the legal procedures |
| **Contact persons and social support** |
| Formal contacts for help-seeking |
| Informal social contact |
| **Information** |
| Corona virus and prevention |
| Testing procedures and isolation in reception centres |
| Procedures and accomodation |
| Test results |
| Other |
| **Well-being/mental health** |
| Self-reported well-being |
| Stressors |
| Factors improving/protecting mental health |
| Other |
| **Corona-related beliefs** |
| Risk perception |
| Severity of SARS-CoV-2 |
| Self-efficacy |
| Making sense of the pandemic |
| Other |
| **Preventive and containment measures** |
| Mask |
| Physical distancing |
| Hygiene |
| Testing |
| Quarantine |
| Mass quarantine |
| Lock-down |
| Other |
| **Assessments, attitude towards measures** |
| **Preventive behaviour** |
| **Daily activities** |
| **Needs** |
| Personal hygiene |
| Room hygiene |
| Social support |
| Internet |
| Information |
| Beverages, special foods, tobacco |
| Medical care |
| Other |
| **Statusposition, hierarchy, dependence** |
| Power relations, dependence due to legal status |
| Limitation/restriction of human rights |
| Respect and dignity |
| (Un)equal treatment |
| Other |
| **Treatment by staff** |
| **Other** |
