# Supplementary material for: Between Care and Coercion: Asylum Seekers’ Experiences With COVID-19 Containment and Mitigation Measures in German Reception Centres
Source: Int J Public Health. 2023 Mar 13;68:1605230. doi: 10.3389/ijph.2023.1605230 (PMC10041458; doi:10.3389/ijph.2023.1605230)
Supplement: Supplementary file 2 [file DataSheet1.docx]

# Supplementary file 1 for the original article:

**“Between Care and Coercion: Asylum Seekers’ Experiences With COVID-19 Containment and Mitigation Measures in German Reception Centres”**

Published in the special issue “Migration health around the globe – a construction site with many challenges” of the International Journal of Public Health

Interview guide. Germany, 2020

How does the Corona virus affect your daily life?

Have you spent time in quarantine because of the Corona virus? *For interviews in quarantine:* How did you come to live in this building?

What does/did a typical day in quarantine/in this building look like for you?

Have you been tested for COVID? How did you come to be tested?

What kind of information did you receive about the Corona virus and about the measures taken?

If you recall the general handling of the Corona virus, the different measures taken and the regulations: All in all, how did you experience the handling of the corona virus here in this accommodation?
